# Plants from a semi-arid environment as a source of phytochemicals against *Fusarium* crown and foot rot in zucchini

**DOI:** 10.1186/s13568-023-01515-0

**Published:** 2023-01-17

**Authors:** Ahmed M. A. Khalil, Ahmed M. Saleh, Sayed M. S. Abo El-Souad, Mahmoud S. M. Mohamed

**Affiliations:** 1 Botany and Microbiology Department, Faculty of Science, Al-Azhar University, Cairo, Egypt; 2 Department of Botany and Microbiology, Faculty of Science, Cairo University, Giza, 12613 Egypt

**Keywords:** Phytochemicals, Polyphenols, Biocides, *Fusarium solani*, Zucchini, Ultrastructure

## Abstract

*Fusarium* crown and foot rot, caused by *F. solani* f. sp. *cucurbitae*, are major fungal diseases affecting zucchini and other cucurbits. Despite the efficacy of synthetic fungicides, their health and environmental hazards have highlighted the urgent need for safer alternatives, such as phytochemical-based biocides. Owing to the upregulation of the plant secondary metabolism under stressful conditions, bioprospecting in harsh environments could reveal ore plants for bioactive metabolites. In this study, thirteen wild plants were collected from their natural habitat in a semiarid environment (Yanbu, Saudi Arabia) and extracted to obtain phenolics rich extracts. Total polyphenols, flavonoids, antioxidant capacities and the antifungal activities of the extracts against a pathogenic isolate of *F. solani* were assessed. *Fusarium solani* was isolated from infected zucchini and characterized by scanning electron microscopy. Hierarchical clustering analysis of the phytochemical screening and in vitro bioactivity revealed that *Rosmarinus officinalis*, *Pulicaria crispa*, *Achillea falcata* and *Haloxylon salicornicum* were the richest in polyphenols and the most powerful against *F. solani*. Further, the extracts of these four plants significantly decreased the disease incidence in zucchini, where *P. crispa* was the premier. Interestingly, results of transmission electron microscopy revealed that extract of *P. crispa*, as a representative of the powerful group, induced ultrastructural disorders in fungal cells. Therefore, this study suggests the use of *R. officinalis*, *P. crispa*, *A. falcata* and *H. salicornicum* grown in semi-arid environments as ore plants to develop phytochemical-based biocides against *Fusarium* crown and foot rot.

## Introduction

*Fusarium* species are the most common soil-born fungi; despite being saprophytes, they are well-known as plant pathogens (Coleman 2016). *Fusarium solani* is a cosmopolitan aggressive plant pathogenic fungus that invades a broad range of host plants (Barreto et al. 2003), and attacks the postharvest crops (Akrami et al. 2012). The diseases caused by *F. solani* are characterized by many symptoms such as root and stem rotting, wilting, leaf yellowing, and sudden death (Nemec et al. 1976). Although the persistence of *F. solani* in the agricultural soil after disease occurrence is poorly investigated, some *formae speciales*, such as *F. solani* f. sp. *cucurbitae* and *fragariae*, have been reported to form chlamydospores that confer survival of phytopathogenic soil borne population after tillage (Henry et al. 2019).

Many researchers demonstrated that *F. solani* f. sp. *cucurbitae* has host specificity for *Cucurbitaceae* (Boughalleb et al. 2007; Paternotte 1987). This phytopathogen has been recorded causing disease in field production in pumpkin, muskmelon, cucumber, watermelon, and rootstock hybrids in addition to be the causal agent of crown rot for several cucurbits including Zucchini squash (Pérez-Hernández et al. 2017). Plant resistance to this pathogen stills limited, while the most common control procedures for the disease caused by *F. solani* f. sp. *cucurbitae* are the rotation with non-host crops, preventive and curative fungicide application. From the human health point of view, application of fungicides is undesirable as low concentrations of synthetic pesticides in agricultural products might cause adverse health effects (Akoto et al. 2013). Further, the accumulation of pesticides in the agriculture soil may lead to irreversible environmental damages such as (i) water-table and water body contamination, (ii) imbalance in biological diversity, (iii) reduce efficiency of microorganisms to degrade pesticides and (iv) develop resistant strains (Boughalleb et al. 2007; Abdelkader et al. 2022).

Despite the worthwhile results of synthetic fungicides against *F. solani*, there is urgent need for developing more safe controlling methods, both for human health and environment (Chandel and Deepika 2010; Mostafa et al. 2009). In this regard, the application of plant bioactive molecules and phytochemicals could provide an effectual alternative for synthetic fungicides (Cowan 1999; Al Kashgry et al. 2020). Among these, phenolic compounds, the most diverse group of plant secondary metabolites including simple phenols, phenolic acids, complex tannins, flavonols and dihydrochalcones, are proved as defensive agents against plant pathogens. Their main advantage is to be environmentally safe as they are easily biodegraded (Steinkellner and Mammerler 2007). Several phenolics are reported to accumulate in plant tissues constitutively (i.e. phytoanticipins) and also hyperaccumulated in response to pathogens challenge (i.e. phytoalexins), playing direct and indirect role in disease resistance (Akhtar and Malik 2000; Lattanzio et al. 2006). Generally, antifungal phenolics could act directly by disrupting the fungal morphology, physiology and ultrastructure (Báidez et al. 2006; Cowan 1999; Khalil et al. 2020; Nguyen et al. 2013) or indirectly by stimulating the plant defense system (Al-Wakeel et al. 2013). The deleterious action of phenolics against fungi could be ascribed to the presence of acidic hydroxyl group attached to aromatic ring that have lipophilic properties allowing phenolic compounds to penetrate the plasma membrane making ionic homeostatic disturbance (Dambolena et al. 2012; Gallucci et al. 2014). Further, these hydroxyl groups could inhibit coupling between the electron transport and phosphorylation reactions (Parvez et al. 2004). Moreover, phenolics have been reported to interfere with the key enzymes that regulate fungal growth and development (Schwalb and Feucht 1999). Therefore, phenolic compounds have been suggested to attend as valuable substitutes to the chemical control of plant pathogens in agricultural soils (Langcake 1981).

From a physiological point of view, the production of biologically active phytochemicals has been reported to be upregulated under adverse environmental conditions (Oh et al. 2009). In this context, due to their severe habitat characterized by little water and high temperature, xerophytic plants have been reported to hyperaccumulate phenolic compounds, rendering them a promising source for antifungal phytochemicals (Fahn and Cutler 1992; Yehia et al. 2020). For instance, *Horwoodia dicksoniae* extracts revealed growth inhibitory effect against *Aspergillus fumigatus*, *Streptococcus pneumoniae* and *Escherichia coli* (Abdelwahab et al. 2016). Moreover, the antifungal activity of *Rhazya stricta* extract was performed against different fungal species including *Trichophyton longifusis*, *Aspergillus flavus*, *Candida albicans* and *F. solani*. It showed a good inhibitory effect with reducing the hyphal growth of all investigated fungal species (Khan and Khan 2007).

Therefore, this study aims to bioprospect in a semi-arid environment (at Yanbu desert, Saudi Arabia) for xerophytes rich in antifungal phenolics, with respect to *F. solani*. Thirteen plants were collected and extracted to obtain phenolics rich extracts.

## Materials and methods

### Isolation and identification of *Fusarium solani*

*Fusarium solani* was isolated in the laboratory from infected basal stems of zucchini plants (*Cucurbita pepo*) displaying external signs of zucchini wilt and crown rot. *Fusarium solani* isolation was achieved by cutting the infected basal stem into small pieces ranging from 2 to 3 mm. The pieces were surface disinfected with sodium hypochlorite (10%) solution for 2 min, washed with sterile distilled water several times. Pieces were transferred aseptically onto *Fusarium* selective medium called modified Nash-Snyder agar (MNSA) (1 g/l KH_2_PO_4_, 0.5 g/l MgSO_4_-7H_2_O, 15 g/l peptone, 20 g/ Agar, 1 g/l pentachloronitobenzen, 0.3 g/l streptomycin sulfate, 0.12 g/l neomycin sulfate) (Nash and Snyder 1962) and incubated at 26 ± 2 °C for 5 days. The fungal hyphae were transferred to petri dishes containing potato dextrose agar medium (PDA). Morphological characteristics were identified both on Czapek-Dox agar (30 g/l sucrose, 3 g/l NaNO_3_, 0.5 g/l KCl, 100 mg/l FeSO_4_-7H_2_O, 0.5 g/l MgSO_4_-7H_2_O, 1 g/l K_2_HPO_4_) and potato dextrose agar (PDA) medium as well as microscopic examination (Booth 1977; Ellis 1976; Raper and Fennell 1965; Raper and Thom 1949; Rifai 1969) To confirm morphological identification, Scanning electron microscopy (SEM) was carried out. It was accomplished to approve the morphological characteristics of fungal species. A small sample of fungal colony was immersed in glutaraldehyde (2.5%) for 15 min. The samples were dehydrated by ethanol-acetone gradient. To avoid collapse in SEM the critical point drying (CPD) was used. Using an Emitech K550X coating unit, the specimens were coated with gold and then mounted into SEM FEI (Quanta 200).

### Pathogenicity test

Pathogenicity of *F. solani* was determined on zucchini plants under greenhouse conditions. To obtain conidial suspensions, the surface of cultures was scratched with a sterile scalpel and then the plate was washed with sterilized distilled water. The inoculum then filtered by gauze to eliminate the large fragments of mycelia. The fungal spores were counted using a hemocytometer and adjusted to an approximate concentration 1.5 × 10^5^ conidial/ml. Root of zucchini seedlings were dipped in conidia suspension while control was carried out by dipping roots in sterile distilled water. The inoculated plants were incubated in greenhouse under 80% relative humidity and 30 ± 2 °C. Koch’s postulates were checked under controlled conditions (Choi et al. 2015).

### Collection of plant materials and preparation of extract

Aerial parts of thirteen desert plants (leaf, petal, pod, seed and stem) were collected from different explored sites in Yanbu region deserts, Saudi Arabia, surrounding the site; 24°6′ 7.2288′N 38°6′ 37.6524′E. The collected plants were washed with tap water followed by distilled water and left in shade and air till completely dried. Each plant sample was separately ground into powder for preparation of the extract. Powdered plant samples were extracted in acetone/methanol (1:1, v/v). After centrifugation at 5000*g* for 15 min, the supernatant was de-fated with n-hexane and then evaporated to dryness with a rotary evaporator. The obtained pellet was dissolved in methanol and used for the subsequent experiments. Unless stated differently, all chemicals and solvents were in analytical grade (Sigma-Aldrich, Milan, Italy).

### Assessment of flavonoids, total phenolics and antioxidant capacity of the plant extracts

The crude extract was used for each plant sample to assess total phenolics by Folin-Ciocalteu method (Kaur et al. 2009). Briefly, 1 ml of the diluted plant extract was mixed with one ml of 10% Folin-Ciocalteu reagent for 3 min. After through, one ml of anhydrous sodium carbonate (20%, w/v) was added to the mixture, followed by incubation in dark for 30 min at room temperature. The absorbance of the mixture was measured at 650 nm. The concentration of phenolics was extrapolated from gallic acid (GA) calibration curve and the results were expressed as mg GAE/ml extract. Total flavonoids contents were assayed by the AlCl_3_ colorimetric method (Sakanaka et al. 2005). Briefly, 50 µl of each crude plant extract was mixed up to 1.45 ml of distilled water, followed by addition of 75 µl of 5% NaNO_2_ solution. The mixture was allowed to stand for 6 min, and then 150 µl of 10% AlCl_3_ solution was added; after 5 min 0.5 ml of 1 mol/l NaOH solution were added. The final volume of the mixture was brought to 2.5 ml with distilled water. The absorbance was measured at 510 nm. A calibration curve of catechin (C) was used to calculate the concentration of total flavonoids and the results expressed as mg CE/ml extract. The total antioxidant capacity (TAC) was assayed by the popular ferric reducing antioxidant power (FRAP) and expressed as µmole Trolox equivalent/g dry weight.

### Evaluation of the inhibitory activities of the desert plant extracts against *F. solani*

#### Inhibition of *F. solani* radial growth

*F. solani* mycelial plugs (5 mm), obtained from seven-days-old culture, were added at the center of plates contain PDA medium (control) or PDA complemented with various amounts of plant extracts. Mycelium radial growth was measured and the inhibitory activity to radial growth was calculated relative to the corresponding control after 14 days. The concentration of extract (mg/ml) needed to bring about 50% inhibition of fungal linear growth. The half maximal inhibitory concentration (IC_50_) was assessed by linear regression.

#### In vivo assessment of the antifungal activity of the most active extracts

Based on the results of the in vitro antifungal experiment, an experiment was conducted to assess the potentiality of the most active extracts in protecting zucchini plants from crown and foot rot disease under greenhouse conditions. To get seedlings, sterilized seeds were sown on previously sterilized peat for three weeks. Seedlings were dipped, by their roots, into the previously prepared conidial suspension 1.5 × 10^5^ conidial/ml of *F. solani* before transplanting to pots containing sterilized peat. Five groups of infected seedlings were prepared, each consisted of 50 plants divided into 10 pots (5/pot). Three days after transplanting the plants in, the first four groups were treated with extract of *Rosmarinus officeniales*, *Pulicaria crispa*, *Achillea falcata* or *Haloxylon salicornicum*, while the fifth group was left without treatment with any plant extract. The extracts were applied to the basal stems of the plants at a concentration equal to the IC_50_, obtained from the results of the in vitro antifungal assay, at the rate of 5 ml/plant. To provide a control, other 50 seedlings were dipped into sterilized distilled water. For each plant of the control group, 5ml of solvent was applied to its basal stem. The pots were kept in the greenhouse under 80% relative humidity and 30 ± 2 °C. Fourteen days after inoculation, disease symptoms started to develop on plants. To estimate disease progress, plants were monitored daily for the presence of the disease symptoms. Four weeks after seedling transplantation, the disease incidence (DI) was calculated as the ratio of symptomatic plants relative to the total number of plants assessed in the experiment. The disease incidence was estimated four weeks after seedling transplant.

#### Transmission electron microscopy (TEM)

According to the results of the in vitro antifungal assay *P. crispa* was selected for TEM investigation. To evaluate the antifungal activity of *P. crispa* extract against *F. solani*, spore suspension (1 × 10^5^ spores/ml) was mixed with 10 ml potato dextrose supplemented with *P. crispa* extract and 2% Tween 20 at half-maximal inhibitory concentration (IC_50_) 0.92 mg/ml. Fungal culture was kept at 25 ± 2 °C under shaking condition (150 rpm) for seven days. Same procedure was prepared to use as a control with free *P. crispa* extract. The mycelia were harvested from the culture media after incubation period and separated by centrifugation at 4000×*g*, 15 min and washed twice using 0.1 M phosphate buffer at pH 7.4. For fixation, the harvested fungal cells were treated by 3% v/v glutaraldehyde for two hours inside the fume hood. Cells were washed with buffer and then re-fixed with 1% (w/v) osmium tetroxide (osmic acid) at 5 °C for three hours. Consequently, cells were dehydrated gradually in a series of ethanol solutions (10%, 20%, 50%, 60%, 70%, 80% and 90%; and 100%). The ultrathin slices (almost 100 nm) were obtained using a Reichert-Jung ultramicrotome with diamond knife. The ultra-sections were put on 200 mesh copper grids and stained with 4% aqueous uranyl acetate/lead citrate to be ready for a JEOL JEM-1400 transmission electron microscope at an accelerating voltage of 40 to 120 kV.

### Statistical analysis

Data analyses were performed using Statistical Analysis System (SPSS Inc., Chicago, IL, USA). Tukey’s Test (*p* < 0.05) was applied for separations of mean (n = 5). Cluster analysis was performed by using Pearson distance metric of the MultiExperiment Viewer (MeV)™ 4 software package (version 4.5, Dana-Farber Cancer Institute, Boston, MA, USA).

## Results

### Isolation and morphological identification of *F. solani*

**Fig. 1:**
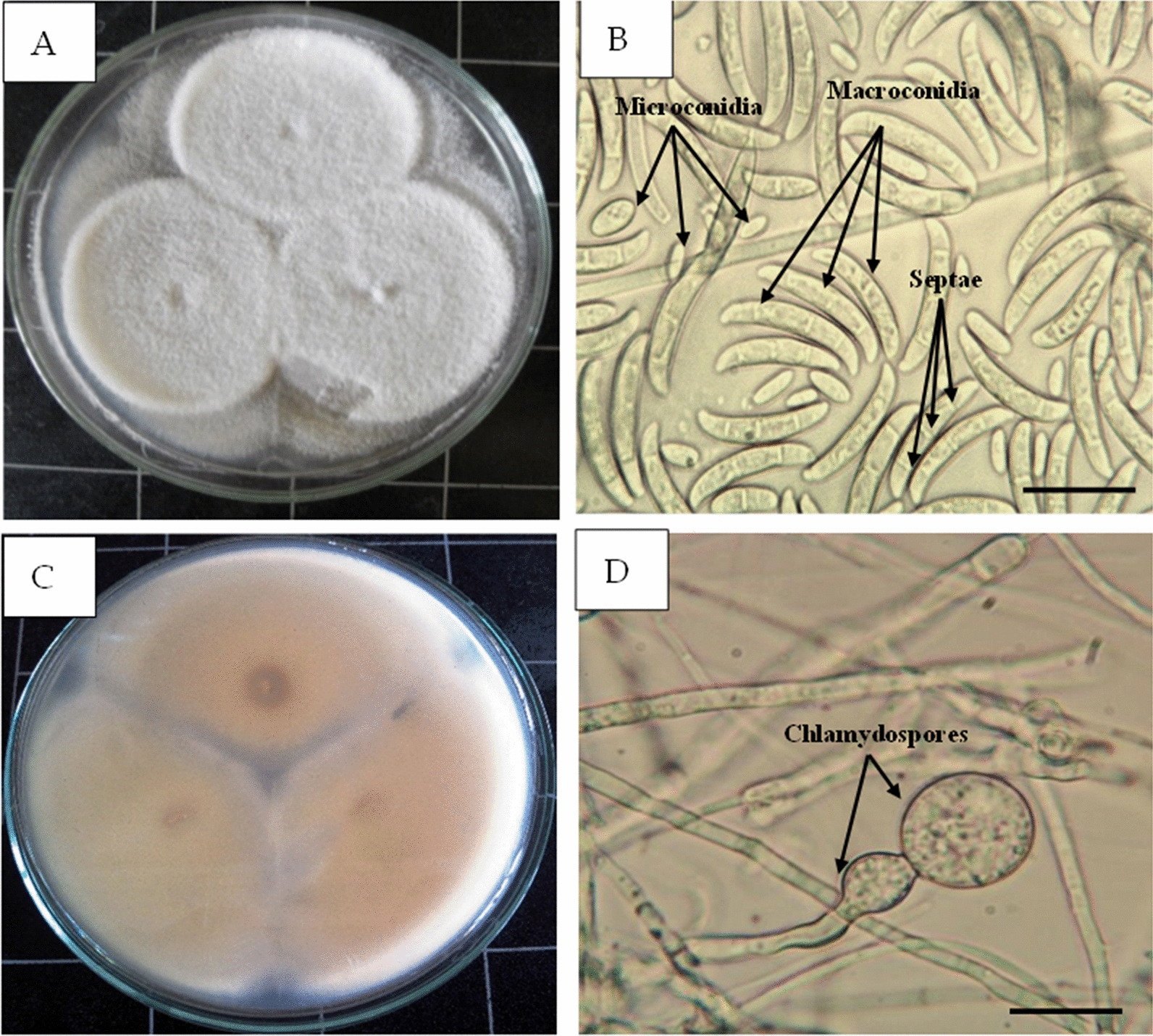
** A** Growth of *Fusarium solani* on potato dextrose agar medium. **B** Bright field micrograph showing microconidia small, oval shape (arrows); macroconidia large and aseptate, canoe-shape with 1–3 septae (arrows) **C** The center and margin color of the reverse petri dish has yellow to tan color. **D** Bright field micrograph showing terminal and sub-terminal chlamidospores. **B** Bar = 1 mm; **D** Bar = 2 mm

**Fig. 2:**
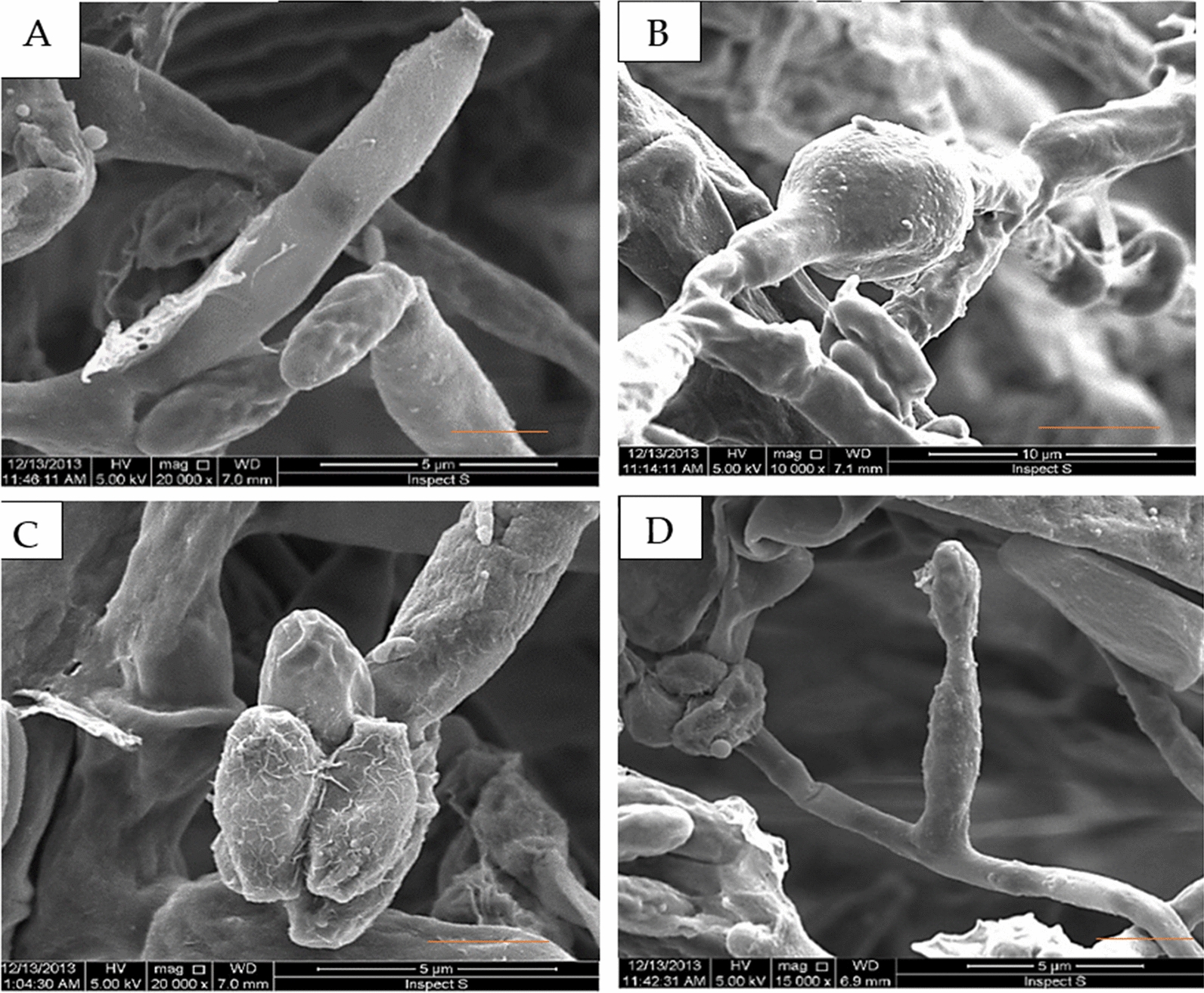
Scanning electron micrographs of *F. solani* showing **A** Solitary conidiophore **B** Chlamidospore **C**, **D** Conidiophores with microconidia. **A**,** C**,** D** Bar = 2.5 μm; **B** Bar = 5 μm

In the present study, isolates were obtained from infected zucchini plants, showing colonies that characterized by cream-to-white color with a yellowish center and a cream or pall yellow reverse on agar (Fig. 1). The *Fusarium* septate hyphae produced non-branched conidiophores terminated with two different types of conidia. Depending on culture age conidia varied in size. Microconidia were oval, unicellular, and abundant, while macroconidia were canoe-shaped, multicellular, septate with one to three septa at an average size of 15.7–28.9 × 2.6–3.4 μm. Chlamydospores were always detected (Figs. 1, 2). The *F. solani* were deposited and available in Culture Collection Ain-Shams University (Cairo-Egypt), under the numbers CCASU-2022-F6.

### Levels of phenolics, flavonoids and TAC show a great variability among of the tested plant extracts

The quantitative evaluation of flavonoids and total polyphenols exhibited considerable variation in their concentrations among the aqueous methanolic extracts of various plant species (Table 1). The levels of total polyphenols ranged from 4.96 to 58.31 mg GAE/ g crude extract and from 1.63 to 24.37 mg CE/g crude extract, respectively. Predictably, the total Phenolic content was higher than that of total flavonoid in all extracts. *Achillea falcata*, member of family *Asteraceae*, showed the highest levels of total phenolics and flavonoids (Table 1). However, the lowest levels of phenolics (4.96 mg/g) and flavonoids (1.63 mg/g) were detected in *A. lachnantha* that belongs to family *Amaranthaceae*. TAC of the tested extracts ranged from 0.33 to 2.8 mM TE/g crude extract. In consistence with the levels of total polyphenols, TAC of *A. falcata* extract was 10-fold higher than that of *A. lachnantha*. These results suggest a clear correlation between the amounts of phenolic compounds in the extracts and their TAC.

**Table 1:** Total phenolics (mg GAE/ml extract), flavonoids (mg CE/ml extract), total antioxidant capacity (TAC, mM TE/g extract) and antifungal activity (IC_50_, mg/ml) of aqueous methanolic extracts of the different plant species

Plant	Phenolics	Flavonoids	TAC	IC_50_
*Horwoodia dicksoniae*	25.08 ± 0.34^e*^	5.14 ± 0.02^d^	0.82 ± .052^c^	1.94 ± 0.05^f^
*Gloiosiphonia capillaris*	14.92 ± 0.53^c^	3.60 ± 0.03^c^	0.59 ± 0.03^b^	2.05 ± 0.04^g^
*Teucrium polium*	29.21 ± 0.13 f^g^	11.44 ± 0.1^g^	1.16 ± 0.03^d^	1.75 ± 0.07^ef^
*Agrostis lachnantha*	4.96 ± 0.96^a^	1.63 ± 0.01^a^	0.33 ± 0.03^a^	1.89 ± 0.16^ef^
*Artemisia sieberia*	40.92 ± 2.51^h^	18.19 ± 0.32^i^	2.32 ± 0.13^h^	1.35 ± 0.05^d^
*Achillea falcata*	58.64 ± 0.30^k^	24.70 ± 0.39^k^	2.81 ± 0.015^i^	0.77 ± 0.03^ab^
*Pulicaria crispa*	42.31 ± 0.78^hi^	17.37 ± 0.22^i^	2.33 ± 0.04^h^	0.67 ± 0.02^a^
*Achillea fragrantissima*	28.22 ± 0.13^f^	7.35 ± 0.1^e^	1.29 ± 0.03^de^	1.51 ± 0.06^de^
*Artemisia Judaica*	30.49 ± 0.08^g^	9.74 ± 0.06^f^	1.33 ± 0.03^e^	1.73 ± 0.04^ef^
*Rosmarinus officinalis*	44.65 ± 0.7^i^	11.09 ± 0.01^ g^	2.80 ± 0.06^i^	0.85 ± 0.02^ab^
*Rhazya stricta*	18.08 ± 0.04^d^	2.46 ± 0.08^b^	0.38 ± 0.2^a^	1.62 ± 0.03^e^
*Haloxylon salicornicum*	41.22 ± 0.02^h^	14.93 ± 0.12^h^	1.86 ± 0.04^f^	1.08 ± 0.04^bc^
*Rhanterium epapposum*	47.22 ± 0.13^j^	22.30 ± 0.25^j^	2.40 ± 0.07^ h^	1.38 ± 0.03^d^

*Values are mean ± standard error of three independent replicates. Different letters in the same column indicate significant difference (*P* > 0.05) as analyzed by Duncan test

### The phenolic rich extracts inhibited the hyphal growth of the tested *F. solani* isolate

**Fig. 3:**
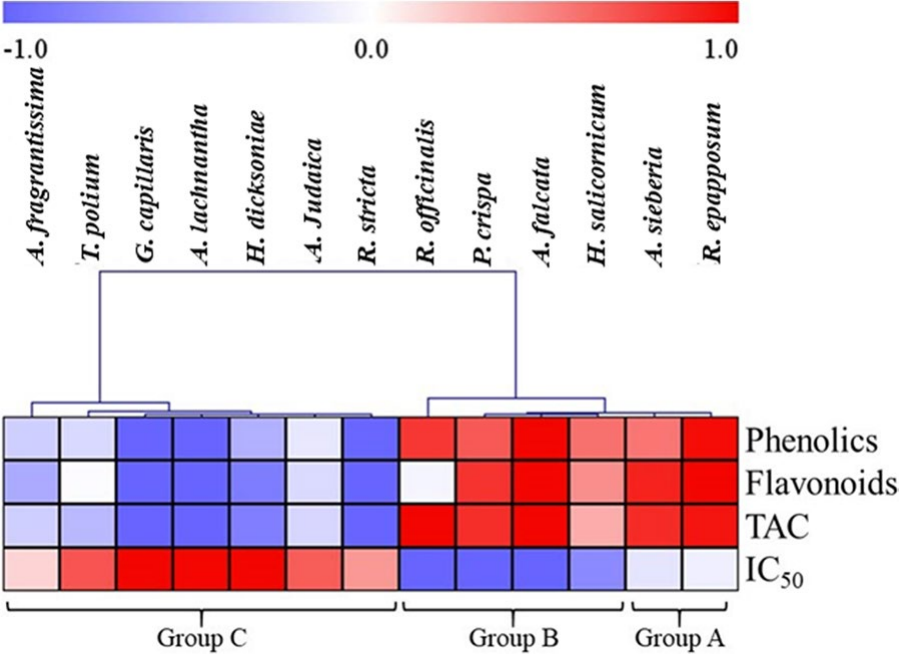
Hierarchical clustering analysis of total polyphenols, flavonoids, total antioxidant capacities (TAC) and antifungal activities (IC_50_) of the different plant extracts. The relative values are shown in the heatmap based on the average value (n = 3) for each metabolite. Red and blue colors indicate lower and higher concentrations, respectively

**Fig. 4:**
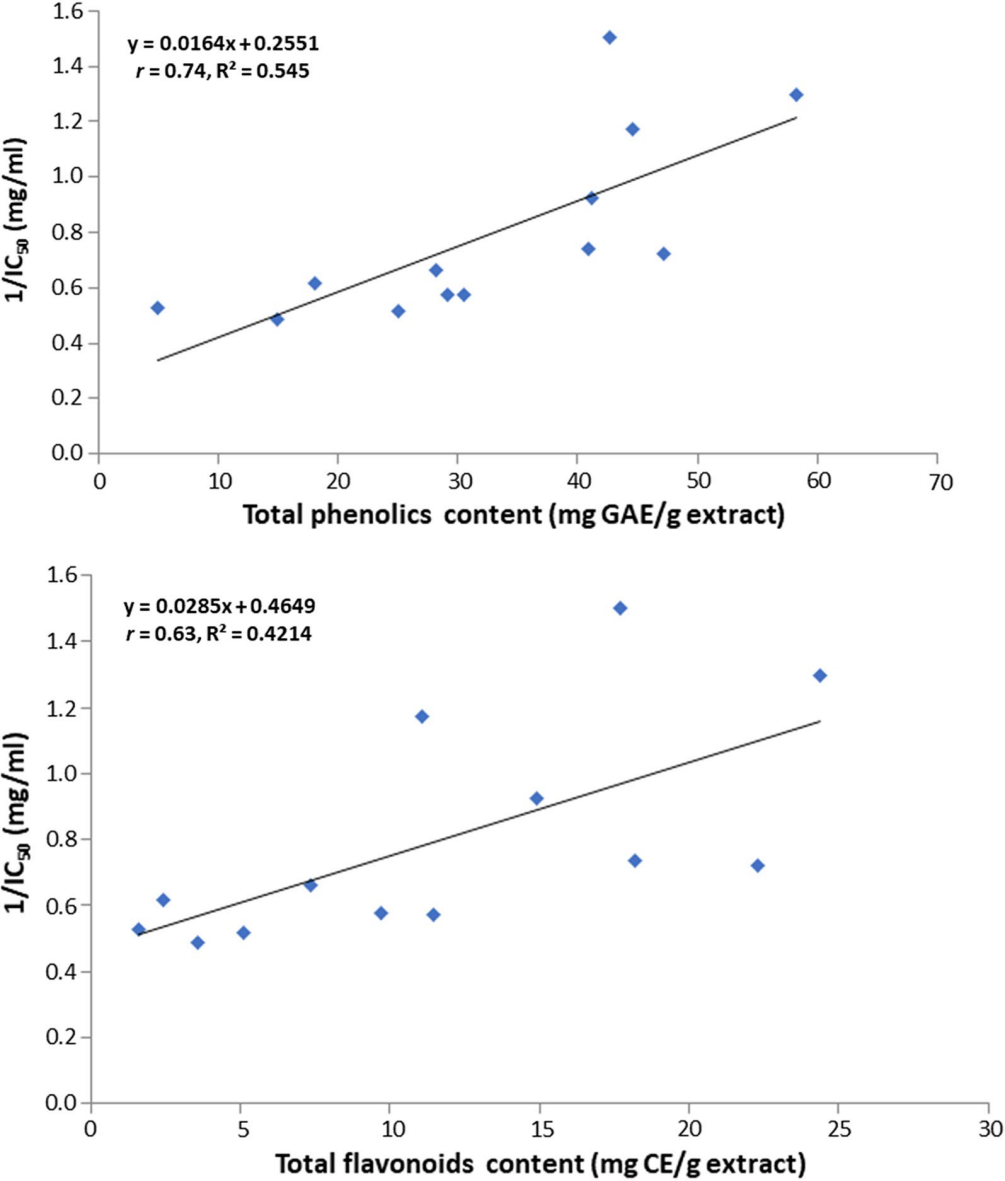
Correlation between the antifungal activity of the plant extracts and their total phenolic (**A**) and total flavonoid (**B**) contents

The antimycotic potential of 13 plant extracts were investigated individually against the investigated *F. solani* isolate. All the tested extracts showed inhibitory effects on the hyphal growth of *F. solani* (Table 1), but at disparate capabilities. According to the results in Table 1 and the hierarchical clustering analysis (HCA, Fig. 3), the crude extracts could be divided into three groups with respect to IC_50_ values against *F. solani*. The groups included extracts characterized by: high antifungal activity (group B; *R. officinalis, P. crispa*, *A. falcata* and *H. salicornicum*); moderate inhibition of *F. solani* (group A; *R. epapposum*, *A. sieberia*); low antimycotic potential (group C; *H. dicksoniae*, *G. Capillaris*, *T. polium*, *A. lachnantha*, *A. fragrantissima*, *A. judaica*, *R. stricta*). Indeed, extracts in groups A and B were characterized by the higher phenolic contents, while those in group C possessed the lower levels of phenolics. This result is consistent with the correlation analysis, where positive relationships were observed between the total phenolics and flavonoids contents of the extract (0.74 and 0.63, respectively) and their antifungal activities (Fig. 4). However, the qualitative variations in the phenolic profiles of the plant extracts and the synergic or antagonistic reactions cannot be neglected (Nwonuma et al. 2019). For instance, although extracts of *P. crispa* and *A. falcata* showed comparable levels of polyphenols but *P. crispa* caused higher reduction in disease incidence.

### *P. crispa*, *A. falcata*, *R. officinalis* and *H. salicornicum* extracts inhibited the development of crown and foot rot disease on zucchini plants

**Fig. 5:**
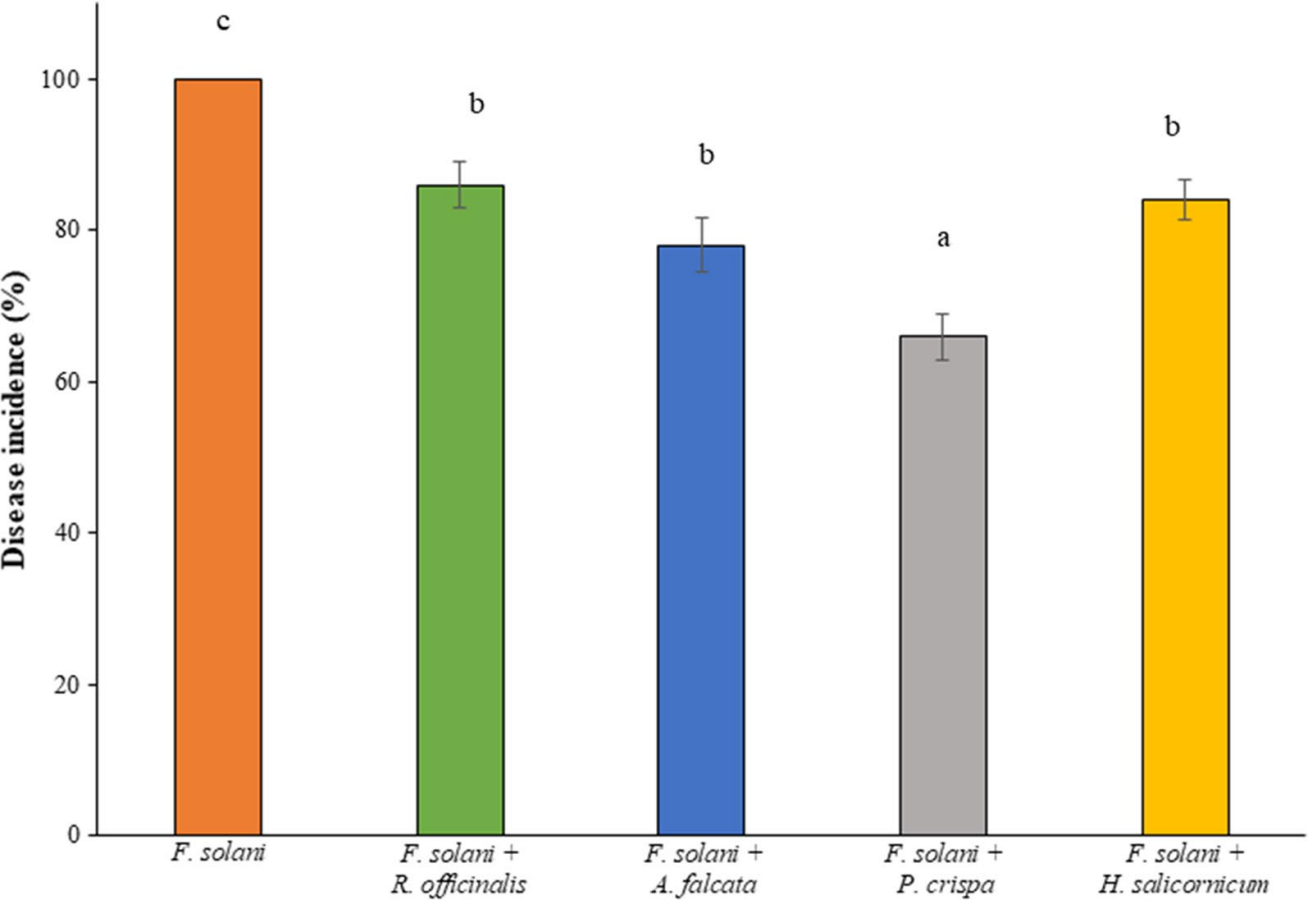
Impact of phenolic rich extracts of *P. crispa*, *A. falcata*, *R. officinalis* and *H. salicornicum* on incidence of Fusarium crown and foot root on zucchini. The different letters above the bars indicate significant differences (*P* < 0.05) as analyzed by the Tukey’s test

Based on the results of the in vitro antifungal assay, the most powerful plant extracts (*P. crispa*, *A. falcata*, *R. officinalis* and *H. salicornicum*) were selected to test their influence on the development of *Fusarium* crown and foot rot on zucchini. Interestingly, the four extracts significantly decreased the disease incidence, where *P. crispa* was the most effective as it resulted of 34% reduction in disease incidence compared to the control (Fig. 5). Although the in vivo activities of phenolic extracts of *P. crispa*, *A. falcata*, *R. officinalis* and *H. salicornicum* against phytopathogens have been rarely investigated, the application of crude extracts of numerous wild plants to reduce the development of plant diseases has been investigated.

### *P. crispa* extract induced ultrastructure disorders in *F. solani*

**Fig. 6:**
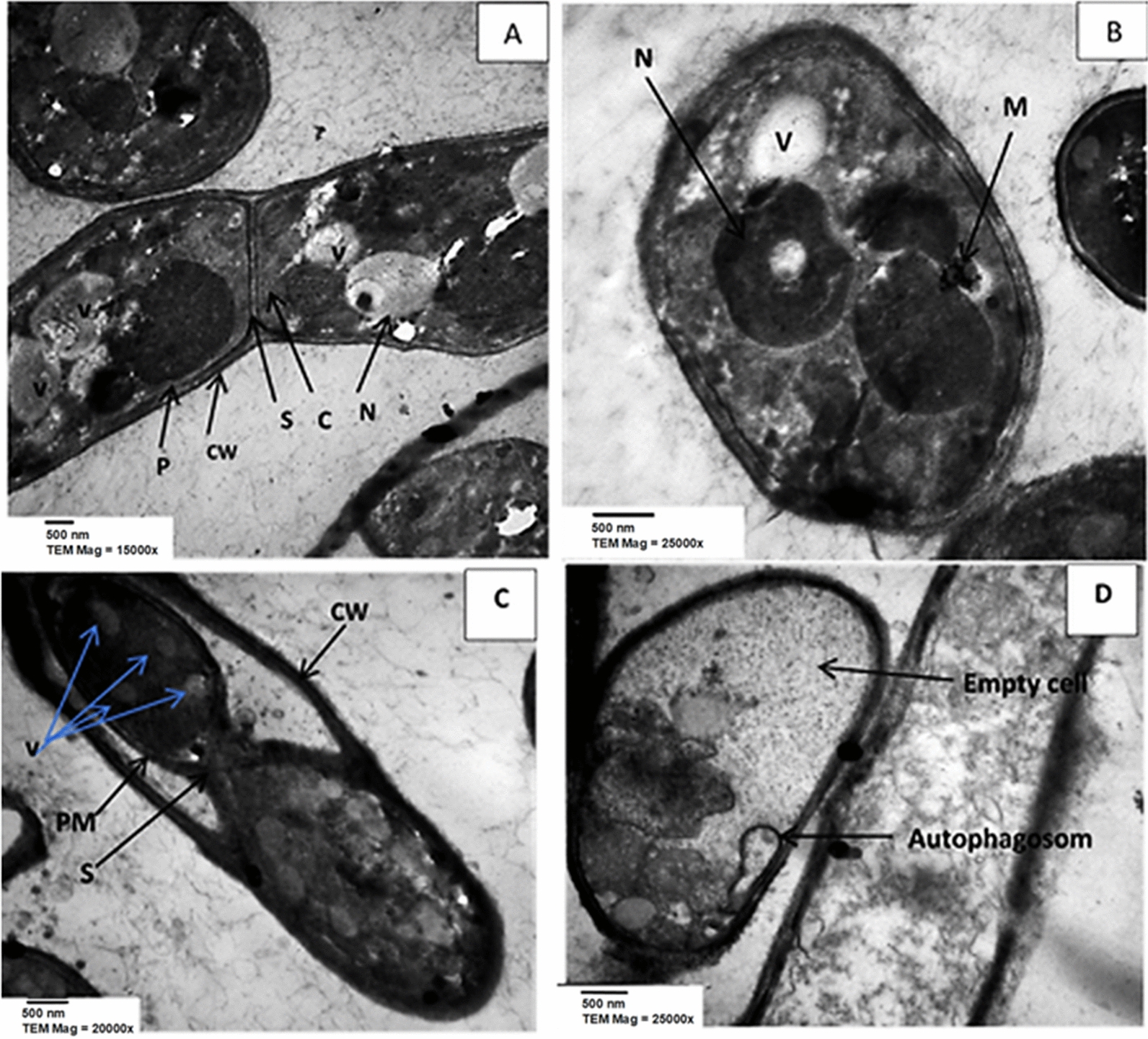
Transmission electron micrographs of *F. solani* growing on PDA medium (**A**, **B**) or amended with 0.92 mg/ml plant extract (**C**, **D**). *CW* cell wall, *V* vacuoles, *PM* plasma membrane, *S* septum, *N* nucleus, *M* mitochondria, *C* cytoplasm

In the present study, transmission electron microscope (TEM) was employed to understand the ultrastructural mechanisms behind the antifungal capacity of the phenolic rich extracts, using *P. crispa* extract as a representative of the most powerful group of extracts (group B, Fig. 3). The hyphae of *F. solani* were observed after treatment with the extract of *P. crispa* in compared to untreated hyphae (control) (Fig. 6A, B). TEM micrographs of hyphal cells of *F. solani* growing on PDA showed a normal, condensed and rigid homogeneous cell wall with plasma membrane closely appraised against the cell wall (Fig. 6A). Cytoplasm appeared dense and metabolically active, determined by the presence of ribosomes and organelles. Moreover, the large number of organelles including mitochondria, vacuoles, nuclei, and endoplasmic reticula found. On the other hand, the observation of hyphal cells of *F. solani* amended on medium containing the extract of *P. crispa* showed various degrees of cell deteriorations (Fig. 6C, D). Obviously, the treatment disturbed plasmalemma, induced intense of cytoplasmic vacuolation while organelles became gradually expanded to the periphery of the cells (Fig. 6C). Autophagosomes were observed that expanded all over the cells (Fig. 6D). Eventually, hyphal cells were highly distorted and crucially damaged where organelles such as mitochondria and nucleus were no longer discernible.

The annual increase in the organic food demand due to consumers awareness of healthy food and environmental save agriculture drive the researchers to explore the natural ecosystem searching for an alternative treatment strategy to chemical pesticide or effective agents to act against phytopathogens. Fungal infection to commercial crops repeatedly leads to high yield worldwide (Savary et al. 2019). In fact, rapid and successful treatment of fungal infection depends on accurate identification of causative agent as well as selection of the suitable treatment strategies. Poletto et al. (2006) had ability to identify *Fusarium* species *via* morphological characters, following procedure suggested by Ohara and Tsuge (2004).

There are several factors such as genetic, morphological and environmental conditions have an influence on plant secondary metabolome, including phenolic compounds, at the qualitative and quantitative levels (Cirak et al. 2014). Alcoholic extracts of 32 plants displayed high variations in phenolic and flavonoid contents (Wojdyło et al. 2007). It was reported that there are many variations in the levels of the total phenolics and flavonoids not only among the plant species but also among the different plant organs (Maisuthisakul et al. 2007; Çirak et al. 2011).

Plant phenolic compounds possess effective free radical scavenging activity (Heim et al. 2002; Maisuthisakul et al. 2007) reported a linear correlation between total phenolic compounds and TAC. It is worth to mention that the potentiality of phenolic compounds as a free radical-scavenging activity depends on their structure, concentration and the substitution of hydroxyl groups (Karamac et al. 2005).

With increasing attention for medicinal and aromatic plants as an alternative medicine, the bioprospecting for bioactive phytochemicals becomes imperative. In this regard, several reports have pointed to the use of natural products, especially phytochemicals, as antifungal agents (Cowan 1999). The antifungal capacity of phenolics against plant pathogenic fungi is well recognized (Almuhayawi et al. 2021; Nguyen et al. 2013). In line with the present results, previous studies have pointed to the significant role of phenolic compounds and their functional groups in the antifungal activity of the crude plant extracts (Wink 2015). The crude extract of *P. crispa* presented strong antifungal activity against *Aspergillus niger* and *Candida albicans* (León et al. 2014). Although the antifungal activity of alcoholic, ethyl acetate and water extracts of several *Achillea* sp. has been reported, the antifungal activity of *A. falcata* phenolic extract is poorly investigated (Zengin et al. 2017). Among 15 wild plants collected from Al-Qassim region, Saudi Arabia, the extract of *H. salicornicum* showed antifungal activities against *Stemphylium botryosum, Alternaria alternata, Botrytis cinerea* and *F. solani* (El-Mergawi et al. 2018). Phenolic rich methanolic leaf extracts of *R. officinalis* collected from Northern Riyadh, Saudi Arabia, showed significant antimycotic properties against *Penicillium ochrochloron*, *P. funiculosum, Aspergillus niger*, *A. flavus A. ochraceus* and *Candida albicans* (Elansary et al. 2020) .

Indeed, the adverse impact of phenolic compounds on fungi depends both on concentration and structure (Ansari et al. 2013; Gallucci et al. 2014). Within this framework, the quantitative structure-activity relationship a strong correlation between the antifungal potential of phenolics and flavonoids and their molecular and structural properties has been revealed (Dambolena et al. 2012; Gallucci et al. 2014). In terms of their mechanisms of action, phenolics and flavonoids can exhibit antifungal activity by disrupting cell division, hyphal formation and / or triggering sever oxidative stress leading to cell death (Ansari et al. 2013; Cowan 1999). This may be attribute to the direct impact of active phytochemicals ingredients on fungal cells or due to induction of defense mechanisms in the host plant (Al-Wakeel et al. 2013; Ansari et al. 2013).

For instance, treatment of tomato seed with *Origanum vulgare* essential oils reduced the incidence of *Fusarium* wilt caused by *Fusarium oxysporum* f.sp. *lycopersici* (Gonçalves et al. 2021). *A*cetone extracts of *Agapanthus caulescens* (15 mg/ml), *Paenibacillus* sp. was reported to significantly inhibit the incidence and severity of black rot, caused by *Xanthomonas campestris*, on the leaves of *Brassica napus* (Mandiriza et al. 2018; Persaud et al. 2019) reported that the application of lemon grass and thick leaf thyme aqueous extracts have reduced the severity of Sheath blight disease caused by *Rhizoctonia solani* in rice. Phenolic extracts of *Orobanche crenata* and *Sanguisorba minor* inhibited the rot of sweet cherry fruits (Gatto et al. 2016). Xerophyte plants showed abroad range of polyphenols including phenolic acids, flavonoids, lignans and coumarins that have an antifungal activity against most of soilborne pathogens and successfully applied as fungicides.

The effects of crude medicinal plant extracts on fungal development, including conidial germination were investigated (Khalil and Hashem 2018; Niño et al. 2012; Ren et al. 2012). Moreover, phenolic compounds including cinnamic, gallic, vanillic, salicylic and ferulic acids are reported to induce collapse and shrinkage of the bacterial and fungal cells (Akter et al. 2019; Nguyen et al. 2013; Abdelmohsen et al. 2020). Supporting this hypothesis, when some plant extracts applied on *Fusarium* species hyphal cells showed similar signs (Benhamou and Thériault 1992). Moreover, some flavonoids such as quercetin and luteolin were induced some morphological and ultrastructural damages of the tested fungal species (Báidez et al. 2006). In line with this concept, the presence of high content of phenolics and flavonoids in plant extracts enable them to disturb the cell permeability, by their lipophilic effects, and uncouple the oxidative phosphorylation due to their hydroxyl groups (Dambolena et al. 2012; Gallucci et al. 2014). It is known that Macroautophagy or type II programmed cell death of fungal cells is activated by constituents of growth medium and associated with cellular stress (Hashem et al. 2020; Khalil et al. 2019). Moreover, autophagosomic-lysosome lyses the bulk cytoplasmic contents, aggregates abnormal protein, and damages cell organelles (Zhang et al. 2016).

In conclusion, studies on xerophyte plant extracts revealed the presence of potent phenolic compounds with inhibitory effects against plant pathogens. *Rosmarinus officinalis*, *Pulicaria crispa*, *Achillea falcata*, and *Haloxylon salicornicum* are phenolic rich plants that prevent Fusarium crown disease and foot rot caused by *F. solani in vitro* and in vivo. The extracts of these four plants significantly reduced the incidence of the disease in zucchini. These plant extracts have a bright future in the field of plant protection to replace traditional synthetic fungicides.

## Data Availability

Not applicable.

## Abbreviations

FRAP: Ferric reducing antioxidant power
MNSA: Modified Nash-Snyder agar
PDA: Potato dextrose agar
SEM: Scanning electron microscope
CPD: Critical point drying
GA: Gallic acid
TCA: Total antioxidant capacity
DI: Disease incidence
TEM: Transmission electron microscope
TAC: Total antioxidant capacity
HCA: Hierarchical clustering analysis
